# Pseudologia Fantastica: a case report

**DOI:** 10.1192/j.eurpsy.2022.1828

**Published:** 2022-09-01

**Authors:** S. Sneep, I. De Jong

**Affiliations:** GGZ Westelijk Noord Brabant, Psychiatry, Halsteren, Netherlands

**Keywords:** pseudologia fantastica

## Abstract

**Introduction:**

Pseudologia fantastica is a psychiatric phenomenon that occurs equally in men and women (1). The condition was first described in 1891 and contains fantasized events (2). Most of the time the fantasized events are not entirely unbelievable. They are based upon blurred fantasy and reality and are stable over time (1,3).

**Objectives:**

We present a case of possible pseudologia fantastica to raise awareness about this phenomenon and possible treatment.

**Methods:**

A literature search in English was performed using Pubmed with the following MeSh terms ‘pseudologia fantastica’.

**Results:**

We present a 20-year old women diagnosed with an intellectual disability (IQ=80) and a post traumatic stress disorder. She received treatment in an outpatient setting for a couple of years. The patient was treated by EMDR therapy and individual therapy sessions. She rejected other forms of therapy or any medication. During the treatment her symptoms were getting worse. The symptoms contained an increase of nightmares and moments of dissociation. The patient was telling she wasn’t able to eat, sleep and function on a daily basis. In individual sessions she reported life-events which worsened over time including; being a victim of rape, seeing her rapist in the subway, being touched and chased by a stranger on her bicycle. Literature search shows that confrontation is one of the treatment methods for this phenomenom.

**Conclusions:**

The treatment of a patient with pseudologia fantastica requires attention for details and acknowledging the possibility of fantasized events, confronting techniques and maintaining an alliance between patient and therapist (2,4).

**Disclosure:**

No significant relationships.

